# Effects of lactic acid bacteria-fermented formula milk supplementation on ileal microbiota, transcriptomic profile, and mucosal immunity in weaned piglets

**DOI:** 10.1186/s40104-022-00762-8

**Published:** 2022-10-06

**Authors:** Ailian Lin, Xiaoxi Yan, Hongyu Wang, Yong Su, Weiyun Zhu

**Affiliations:** 1 Jiangsu Key Laboratory of Gastrointestinal Nutrition and Animal Health, College of Animal Science and Technology, Nanjing Agricultural University, Nanjing, 210095 Jiangsu China; 2 National Center for International Research On Animal Gut Nutrition, Nanjing Agricultural University, Nanjing, 210095 China

**Keywords:** Lactic acid bacteria-fermented formula milk, Microbiota, Mucosal immunity, Transcriptomic profile, Weaned piglet

## Abstract

**Background:**

Lactic acid bacteria (LAB) participating in milk fermentation naturally release and enrich the fermented dairy product with a broad range of bioactive metabolites, which has numerous roles in the intestinal health-promoting of the consumer. However, information is lacking regarding the application prospect of LAB fermented milk in the animal industry. This study investigated the effects of lactic acid bacteria-fermented formula milk (LFM) on the growth performance, intestinal immunity, microbiota composition, and transcriptomic responses in weaned piglets. A total of 24 male weaned piglets were randomly divided into the control (CON) and LFM groups. Each group consisted of 6 replicates (cages) with 2 piglets per cage. Each piglet in the LFM group were supplemented with 80 mL LFM three times a day, while the CON group was treated with the same amount of drinking water.

**Results:**

LFM significantly increased the average daily gain of piglets over the entire 14 d (*P* < 0.01) and the average daily feed intake from 7 to 14 d (*P* < 0.05). Compared to the CON group, ileal goblet cell count, villus-crypt ratio, sIgA, and lactate concentrations in the LFM group were significantly increased (*P* < 0.05). Transcriptomic analysis of ileal mucosa identified 487 differentially expressed genes (DEGs) between two groups. Especially, DEGs involved in the intestinal immune network for IgA production pathways, such as polymeric immunoglobulin receptor (*PIGR*), were significantly up-regulated (*P* < 0.01) by LFM supplementation. Moreover, trefoil factor 2 (*TFF2*) in the LFM group, one of the DEGs involved in the secretory function of goblet cells, was also significantly up-regulated (*P* < 0.01). Sequencing of the 16S rRNA gene of microbiota demonstrated that LFM led to selective enrichment of lactate-producing and short-chain fatty acid (SCFA)-producing bacteria in the ileum, such as an increase in the relative abundance of *Enterococcus* (*P* = 0.09) and *Acetitomaculum* (*P* < 0.05).

**Conclusions:**

LFM can improve intestinal health and immune tolerance, thus enhancing the growth performance of weaned piglets. The changes in microbiota and metabolites induced by LFM might mediate the regulation of the secretory function of goblet cells.

**Supplementary Information:**

The online version contains supplementary material available at 10.1186/s40104-022-00762-8.

## Background

To improve the reproductive efficiency of sows, weaning of piglets in commercial pig farms is usually advanced within three to four weeks after birth and involves a sudden dietary transition from maternal milk to solid food [1]. Therefore, early weaning is usually accompanied by gastrointestinal tract development hysteresis, functional impairment, and gut microbiota dysbiosis, represented by reduced digestibility, diarrhea, intestinal inflammation, and poor growth, generally referred to as the weaning stress syndrome [2]. The concerns about weaning stress and its concurrent symptoms have always aroused great caution in the animal industry. Previous work demonstrated that the gut microbiota of mammals has a large number of roles benefiting the host–such as digestion and fermentation of carbohydrates [3], production of vitamins [4], maintenance of normal functions of the intestinal villi [5], regulation of the immune responses [6], and protection from pathogenic bacteria [7]. However, the gut microbiota of piglets is a complex ecosystem showing dynamic composition and diversity, which could change with age and diet [8]. The transition from sow’s milk to solid feed in early weaning may induce the gut microbiota dysbiosis in piglets [9], which emerges as a leading cause of post-weaning diarrhea [10].

One of the most influencing strategies for the maintenance and modulation of gut microbiota is the oral administration of particular microbes, which are known as probiotics with a definition of “live microorganisms that when administered in adequate amounts confer a health benefit on the host” [11]. Of note, the food matrix could augment or wane the potential effects of probiotics. Therefore, it is paramount to consider the nature of food matrixes, in which probiotics are carried before their intervention [12]. As we all know, fermented milk is a dairy product with a good prospect for incorporating probiotic cultures due to its specific chemical and physical characteristics, including low pH value, high buffering capacity, nutritional content, and watery texture [13]. These conditions facilitate survivability of probiotic strains and tolerance to the low pH conditions encountered during gastric transit. Despite that plenty of in vivo functionalities of LFM has been confirmed and recognized, the application of LFM in the swine breeding industry still lacks extensive investigation. Therefore, in this study, we evaluated the effect of supplementing fermented milk with probiotic LAB isolated from fresh swine feces on the intestinal health of weaned piglets.

Early weaning is prone to high gastric pH and insufficient digestive enzymes such as pepsin, decreasing nutrient digestibility. Acidifiers as additives can reduce intestinal pH, promote intestinal health of weaned piglets, and support the colonization of LAB in the gut of piglets [14]. On the other hand, studies have shown that breast milk is rich in oligosaccharides that shape the infant microbiome [15–17]. Thus, infant formula is often supplemented with prebiotics, such as galactooligosaccharides, fructooligosaccharides, and polydextrose, which are structurally different from breast milk but can mimic the anti-inflammatory activity to a certain extent and benefit the gut microbiota development [18, 19]. Many studies have also demonstrated the positive effects of oligosaccharides in promoting piglet health [20, 21]. Thus, we add appropriate amounts of acidulants and oligosaccharides as additives into the LFM to promote the establishment of beneficial microflora in the gut of weaned piglets. This study was conducted to evaluate the effects of fermented milk on the growth performance, intestinal development, intestinal microbial structure, and immune function of piglets within two weeks after weaning. It revealed a possible mechanism underlying the effects of probiotic fermented milk on promoting the intestinal health of weaned piglets.

## Materials and methods

### Preparation of LFM

In our previous study, three strains (*Enterococcus faecium* LAB8, *Enterococcus hirae* LAB20, and *Lactobacillus johnsonii* LAB19) with probiotic potential were selected out of 21 LAB strains isolated from fresh swine feces, depending on the acidifying activity and tolerance to bile salts and acids. These LAB species were previously reported to be safe and beneficial to host health [9, 22, 23]. LFM was co-fermented by the three strains with 15% bovine milk powder (milk protein 24.92%, milk fat 24.00%, lactose 43.08%), functional oligosaccharides (1.5% oligosaccharides and 0.5% galactose), and 8% sucrose as substrates. The substrates were dissolved with sterile water in 3-L glass jars and pasteurized after stirring. Then the three strains were inoculated into the pasteurized liquid milk for fermentation at 37 °C for 12 h, shaking well the LFM per 2 h during fermentation. The fermented milk was mixed with 0.25% compound acidifier (comprised of 40% lactate, 14% calcium formate, 10% citric acid, 8% caprylic acid, and 28% emulsifier) to adjust the pH value of fermented milk to 3.8–4.0. The finished LFM was stored at 4 °C after sterile packaging. Before animal trial, LAB colony forming units (CFU) in LFM was determined as 7.50 × 10^8^ CFU/mL by the plate count method on MRS agar plates.

### Animals and experimental design

This study was approved by the Nanjing Agricultural University Animal Care and Use Committee (Nanjing, Jiangsu Province, China) (SYXK2019-0066). All animal care procedures in the experiment were operated according to the standard of Experimental Animal Care and Use Guidelines of China (EACUGC2018-01). Every two piglets were housed in metal floor cages (height, 0.85 m; length, 1.2 m; width, 0.70 m) in a suitable environment (20–25 °C, 50%–65% RH). After a 2-day adaptation period, a total of 24 healthy male weaned piglets [(Landrace × Large White), 30 days of age, 9.83 ± 0.18 kg] were randomly divided into two groups with similar body weight at d 0 of this experiment, each group consisted of 6 replicates (cages) with 2 piglets per cage. As reported in previous studies, a reasonable supplemental level of fermented milk for piglets is 130 mL to 250 mL per day [24–26]. Therefore, each piglet in the LFM group were supplemented with 80 mL LFM three times a day at 7:00, 12:00, and 17:00, respectively. Piglets in the CON group were treated with the same amount of drinking water at the same time as the LFM group. The supplemental LFM or water was offered individually to the piglets via a small feeding trough, and it was removed immediately after drinking up (within 1 min). All pigs were fed with a corn-soybean based diet (Additional file 1: Table S1) at fixed times (7:00, 12:00, and 17:00) daily and fed ad libitum (allow for 5%–10% orts on an as-fed basis) during the whole experimental period. High hygiene standards were always maintained to prevent bacterial cross-contamination between the different cages. Individual fasting body weight was registered at 6:00 on the d 0, 7 and 14 of this experiment to calculate average daily gain (ADG) per cage. The feed intake of piglets in each cage was recorded weekly for calculation of average daily feed intake (ADFI) and feed/gain ratio (F/G, expressed as ADFI:ADG).

### Sampling

One piglet was randomly selected from each replicate cage for euthanasia after 12 h fasting at d 14 of this experiment. Euthanasia was performed immediately with jugular vein injection of 4% sodium pentobarbital solution (40 mg/kg) after carefully moving piglets from the cages to the adjacent slaughter area, and all animal samples were rapidly collected within 2 h. After exsanguination, the entire gastrointestinal tract was divided into stomach, duodenum, jejunum, ileum, cecum, and colon, according to the anatomical line [27]. The duodenum was distinguished by the pylorus and duodenal jejunal flexure. The jejunum was separated from the ileum by the ileocecal fold. The terminal end of the ileum was the ileocecal orifice, the junction of the cecum and colon. The length and weight (with contents) of each intestinal segment were measured, and intestine weights were converted to intestine indices by dividing their final body weight. The 2-cm intestinal segment (with digesta) at the midpoint of ileum was removed and fixed in 20 mL 10% (v:v) phosphate-buffered formalin solution for histologic study. After pH measurement (pH 300, HANNA Instrument, Padova, Italy), the digesta of the remaining ileum samples was collected in a sterile tube and stored at – 80 °C for subsequent SCFA and lactate assays. Afterward, the ileum segments were cut longitudinally along the intestine and rinsed with sterile saline. Mucosal samples were carefully collected by scraping the luminal surface with a sterile glass slide and immediately stored in a sterile tube at – 80 °C for further gene expression analysis.

### Analysis of intestinal morphology and goblet cells enumeration

The procedures for hematoxylin and eosin (HE) staining and alcian blue-periodic acid-shiff (AB-PAS) were based on a previous study [28]. In short, the ileum samples were fixed in formalin for 48 h and then were dehydrated in a graded series of ethanol solutions and embedded in paraffin wax. Paraffin-embedded ileum tissues were cool at – 20 °C and cut into 4 μm thick slices by a rotary microtome (RM2235, Leica, Wetzlar, Germany) with a 5° clearance angle. The obtained slices were stained with the HE Stain Kit and AB-PAS Stain Kit, respectively, according to the manufacturer’s protocol (Jiancheng Bio Ins., Nanjing, Jiangsu, China). The stained slides were observed under the ECLIPSE E100 microscope (Nikon Instruments Inc., Shanghai, China) and measured using the Mshot Image Analysis System V1.0 software (MSHOT, Guangzhou, China). For each sample, a total of thirty intact villi from 5–6 photomicrographs of one slide were randomly measured to calculate the average of the villus height (VH) and crypt depth (CD), villus-crypt ratio (VH/CD), and the enumeration of goblet cells.

### DNA preparation and 16S rRNA gene sequencing

According to a suggested method in the previous study, the thawed ileal digesta were digested with 1 mg/mL Protease K (Sigma, St Louis, MO, USA) in a buffer containing 50 mmol/L Tris (pH 7.5), 100 mmol/L EDTA, and 0.5% SDS for 5 h at 55 °C. Ileal microbiota DNA was then isolated by phenol–chloroform extraction following ethanol precipitation [29]. The concentration of extracted DNA was determined by a Nano-Drop spectrophotometer (Thermo, Wilmington, DE, USA). The V4-V5 region of the bacteria 16S rRNA gene was amplified by polymerase chain reaction (PCR) using primers 515F (5’-barcode- GTGCCAGCMGCCGCGG-3’) and 907R (5’-CCGTCAATTCMTTTRAGTTT-3’), of which the reactions conditions were consistent with previous reports [30]. PCR products were extracted from 2% (w/v) agarose gels and purified using the AxyPrep DNA Gel Extraction Kit (Axygen Biosciences, Union City, CA, USA) according to the manufacturer’s instructions. Then, Purified PCR products were quantified by Qubit®3.0 (Invitrogen, Carlsbad, CA, USA) and used to construct the pair-end library following Illumina’s genomic DNA library preparation procedure [31]. The raw reads were deposited into the Sequence Read Archive database under the accession number: SRP365994.

### Lactate and SCFA measurement

Short-chain fatty acid concentrations in ileal digesta were measured by gas chromatography as a described method in our previous study [30]. Briefly, the mixture of 0.4 ± 0.01 g of ileal digesta and 1.6 mL of sterile double distilled water was centrifuged (13,000 × *g*) for 10 min at 4 °C. Transferred 1 mL of supernatant to a new tube and mixed with 0.2 mL of 25% (w/v) metaphosphoric acid. The thoroughly mixed samples were stored at – 20 °C for 12 h to precipitate proteins. After thawing, the samples were centrifuged at 13,000 × *g* for 10 min to obtain supernatants. Then the supernatants were filtered with 0.22 µm syringe filters and analyzed on an Agilent 7890B system (Agilent, Palo Alto, CA, USA). The lactate concentration in the ileal digesta was determined with the enzymatic colorimetric method according to the instructions of the Lactate Assay Kit (Jiancheng Bio Ins., Nanjing, Jiangsu, China). Absorbance was measured at 570 nm using the Olympus AU2700 auto analyzer (Olympus, Tokyo, Japan). All analyses were performed in triplicate to calculate the average.

### Assessment of immunological parameters

Ileal mucosa samples were rapidly ground into powder in cryogenic liquid nitrogen and stored at − 80 °C. For detecting the levels of secretory immunoglobulin A (sIgA), interleukin 10 (IL-10), interleukin 6 (IL-6) and tumor necrosis factor-α (TNF-α), the tissue suspension was prepared by diluting 1 g of ileal mucosa samples 1:9 (w/v) with PBS buffer (0.01 mol/L, pH 7.2–7.4) to meet the detection limit of the commercial kits. The immunological parameters were determined using the Fankew Porcine ELISA Kits ( FANKEL Industrial Co., Ltd., Shanghai, China) and normalized by the the total protein concentration to determine the concentration of sIgA or cytokine per mg protein for each sample. The total protein concentration of tissue suspension were determined by the BCA Protein Assay Kit (Jiancheng Bio Ins., Nanjing, Jiangsu, China). The total protein and immunological parameter assays were manually performed in triplicate by a multichannel pipette and measured the absorbance with Olympus AU2700 auto analyzer (Olympus, Tokyo, Japan).

### Library construction of RNA-Seq data and real-time PCR validation

Total RNA was extracted from ileal mucosa using TRIzol Reagent (Invitrogen, Carlsbad, CA, USA) following the manufacturer’s protocol. The integrity and purity of extracted RNA were detected by Bioanalyzer 2100 (Agilent, Palo Alto, CA, USA) with OD260/280 > 1.8 and RIN number > 7.0. Because there were six replicate cages from each group, four biological replicates (one piglet in each replicate cage) were randomly selected for the RNA-Seq to reduce the costs of the experiment. Poly(A) RNA was purified from 1 μg total RNA after two rounds of purification by Dynabeads Oligo (Thermo, Wilmington, DE, USA) and was reverse-transcribed to the final cDNA library with a Reverse Transcriptase (Invitrogen, Carlsbad, CA, USA), according to the manufacturer’s procedure. The paired-end sequencing was performed on the Illumina Novaseq™ 6000 platform (LC Bio Technology Corporation, Hangzhou, Zhejiang, China), following the vendor’s recommended protocol [32]. The RNA-seq datasets were submitted to the Sequence Read Archive database under the accession number: SUB11204174. The cDNA for real-time PCR validation was rever se-transcribed from total RNA by the Reverse Transcription Kit (Vazyme, Nanjing, Jiangsu, China). The PCR reactions of cDNA were performed in a 20-μL mixt ure at the Quant-Studio Step One Plus™ system (Thermo, Wilmington, DE, USA) following the instructions of the SYBR Green qPCR Kit (Vazyme, Nanjing, Jiangsu, China). The sequences of primers were designed using Primer-BLAST on the National Center for Biotechnology Information website (Additional file 1: Table S2). Each cDNA had performed triplicate PCRs to calculate the average threshold cycle (Ct) with the porcine β-actin gene as the reference, and the relative expression of target gene mRNA was calculated with the formula 2^−ΔΔCt^ [33]. Finally, for the convenience of comparison, relative quantification values from real-time PCR and fragments per kilobase per million mapped fragments (FPKM) values from the transcriptomic data were presented as fold change (FC) [34].

### Data analysis 

Data were analyzed by SPSS 24.0 as a randomized block design, considering LFM as the main effect and the replicate as a block. Cage was regarded as one experimental unit (*n* = 6) of all analyses except body weight and ADG, which were tested for significance along time using the Mixed Model with LFM treatment and cage as within-subject factor. The Student’s *t*-test was used to evaluate the significance of differences in other variables between the two groups because only one piglet per cage was sampled and represented the cage mean. All values are presented as group means and standard error of the mean (SEM). The difference at *P* < 0.05 was considered statistically significant, while a difference at 0.05 ≤ *P* < 0.10 were considered a trend.

Power analyses by R package “micropower” [35] calculated before the start of the experiment identified a sample size of *n* = 6 could obtain a power greater than 0.9 with an ω^2^ = 0.12, given with a type I error of 5% based on a PERMANOVA test using the Bray–Curtis beta metric. Regarding microbiota profiling, raw FASTQ files were first demultiplexed, quality-filtered by Trimmomatic, and merged by FLASH as the criteria described previously [36, 37]. Sequences were clustered into operational taxonomic units (OTUs) at 100% similarity using the Deblur denoising algorithm to remove noise of error sequence [38]. OTUs were clustered with a 97% similarity cutoff using UPARSE (version: 7.1) [39]. The Chao 1, ACE, Shannon, and Simpson diversity indices were used to reflect α diversity at 97% identity and performed using Mothur software (version: 1.35.03) [40]. Principal coordinate analysis (PCoA) was conducted based on the Bray–Curtis distance. For identifying biomarkers in ileum microbiota, linear discriminant analysis effect size analysis (LEfSe) was performed by an online LEfSe algorithm [41]. The algorithm uses the Kruskal–Wallis sum rank test to examine features with significant differential abundance, followed by linear discriminant analysis (LDA) to screen the effect size of each distinctively abundant taxa (i.e., LDA score > 2, *P* < 0.1) [42].

According to a reported power analysis method for RNA-Seq [43], the sample size of 4 could achieve a power of 0.92 with the high-sequencing depth of 36 M reads/replicate and false discovery rate (FDR) cutoff of 0.05. The raw transcriptomic sequencing was converted into clean reads by Seqtk [44], then based on the quantitative results of clean reads mapping to referential pig genome (Sus scrofa 10.2) with Hisat2 (version: 2.0.4), the gene expression levels were normalized by presenting as FPKM and FC [45]. Genes with altered expression at the particular screening criterion (*P* < 0.05, |log_2_ (FC)|> 1) were selected by the edgeR for the further analysis [46]. Gene Ontology (GO) functional enrichment, genetic network analysis, and Kyoto Encyclopedia of Genes and Genomes (KEGG) pathways analysis were respectively performed using OmicStudio [47], Metascape [48], and KOBAS [49] to identify potential genes and pathways associated with LFM treatment.

## Results

### Growth performance and digestive organ indices

**Fig. 1:**
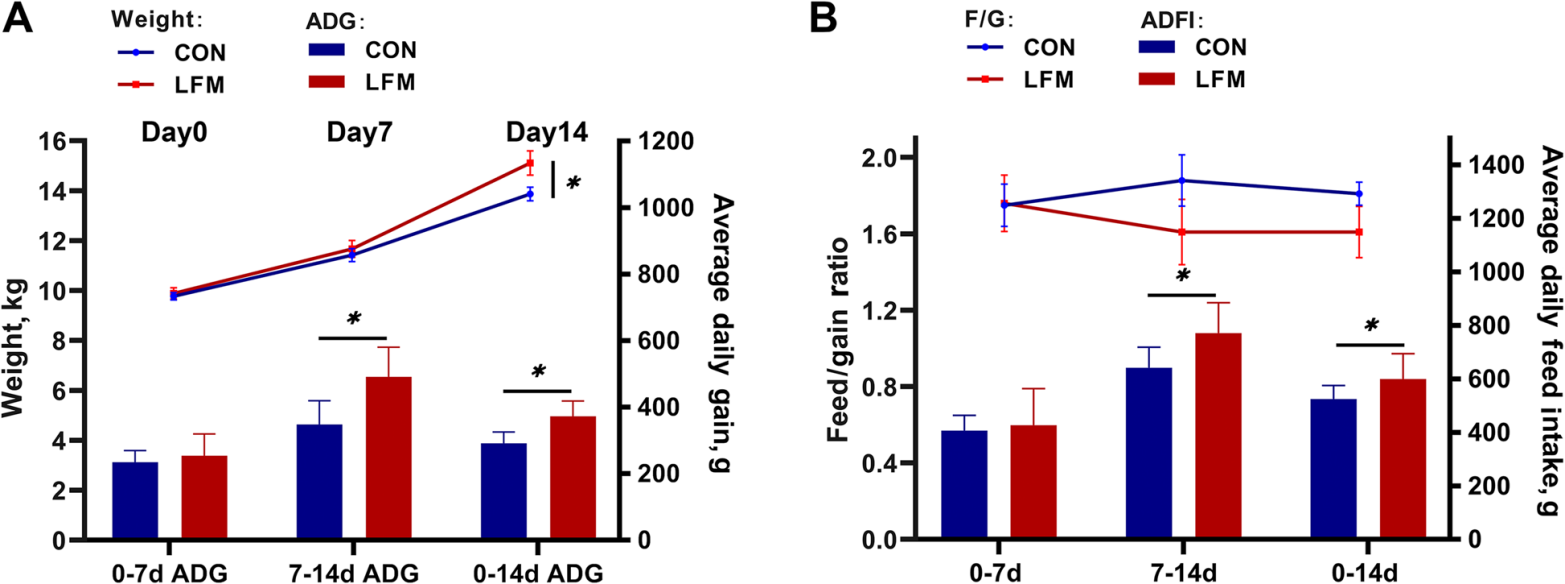
Effects of LFM on growth performance of weanling piglets. **A** The change in weight gain of weaned piglets. **B** The feed efficiency of weaned piglets. Data are mean ± SEM. *P < 0.05, **P < 0.01, ***P  < 0.001

All piglets kept healthy and had no diarrhea throughout the experiment. The final body weight, ADG, and ADFI in the LFM group were significantly higher than that of the CON group (*P* < 0.05) during the whole experimental period (Fig. 1). From d 7 to d 14, LFM treatment increased ADG and ADFI compared with the CON group (*P* < 0.05). There was no significant difference in F/G between the two groups during the experiment (*P* > 0.05). The organ indices of the jejunum, colon, small intestine, and total intestinal tract in the LFM group were higher than that of the CON group (*P* < 0.05), while the organ indices of the ileum (*P* = 0.06) had an increasing tendency compared with the CON group (Table 1). Analogously, LFM treatment significantly increased the lengths of the jejunum, small intestine, and total intestinal tract (*P* < 0.05). Besides, ileum length (*P* = 0.06) in the LFM group had an increased tend compared with the CON group.

**Table 1:** Effects of LFM on macroscopic intestine organ parameters of weaned piglets^a^

Item	CON	LFM	*P*-value
Body weight, kg	13.86 ± 0.42	15.11 ± 0.62	0.002
Intestine indices^b^, %			
Stomach	0.96 ± 0.17	0.97 ± 0.21	0.931
Duodenum	0.13 ± 0.03	0.13 ± 0.01	0.827
Jejunum	4.41 ± 0.37	5.11 ± 0.49	0.020
Ileum	0.26 ± 0.01	0.29 ± 0.02	0.062
Cecum	1.17 ± 0.21	0.99 ± 0.18	0.152
Colon	2.56 ± 0.24	2.96 ± 0.29	0.031
Small intestine	4.81 ± 0.39	5.54 ± 0.49	0.018
Large intestine	3.74 ± 0.25	3.96 ± 0.31	0.229
Total gastro-intestinal tract	9.52 ± 0.51	10.48 ± 0.41	0.005
Intestine length, cm			
Stomach	13.5 ± 1.9	15 ± 1.8	0.209
Duodenum	28.5 ± 2.2	29.6 ± 0.8	0.262
Jejunum	1274.1 ± 30.6	1367.3 ± 48.2	0.003
Ileum	50 ± 4.8	55.1 ± 3.3	0.056
Cecum	12.8 ± 2.4	14.5 ± 2.8	0.302
Colon	225.8 ± 12.9	236.5 ± 17.2	0.253
Small intestine	1352.3 ± 35.0	1452.1 ± 46.8	0.002
Large intestine	238.6 ± 12.3	251.1 ± 16.1	0.167
Total gastro-intestinal tract	1591.3 ± 47.3	1703.1 ± 48.7	0.002

^a^Values are presented as mean ± SEM, *n* = 6; *CON*, a control group; *LFM*, a lactic acid bacteria-fermented formula milk supplementation group

^b^Intestine indices refer to the ratio of Intesti nal organ weight to the final body weight

### Morphological and immunological parameters in ileal mucosa

**Fig. 2:**
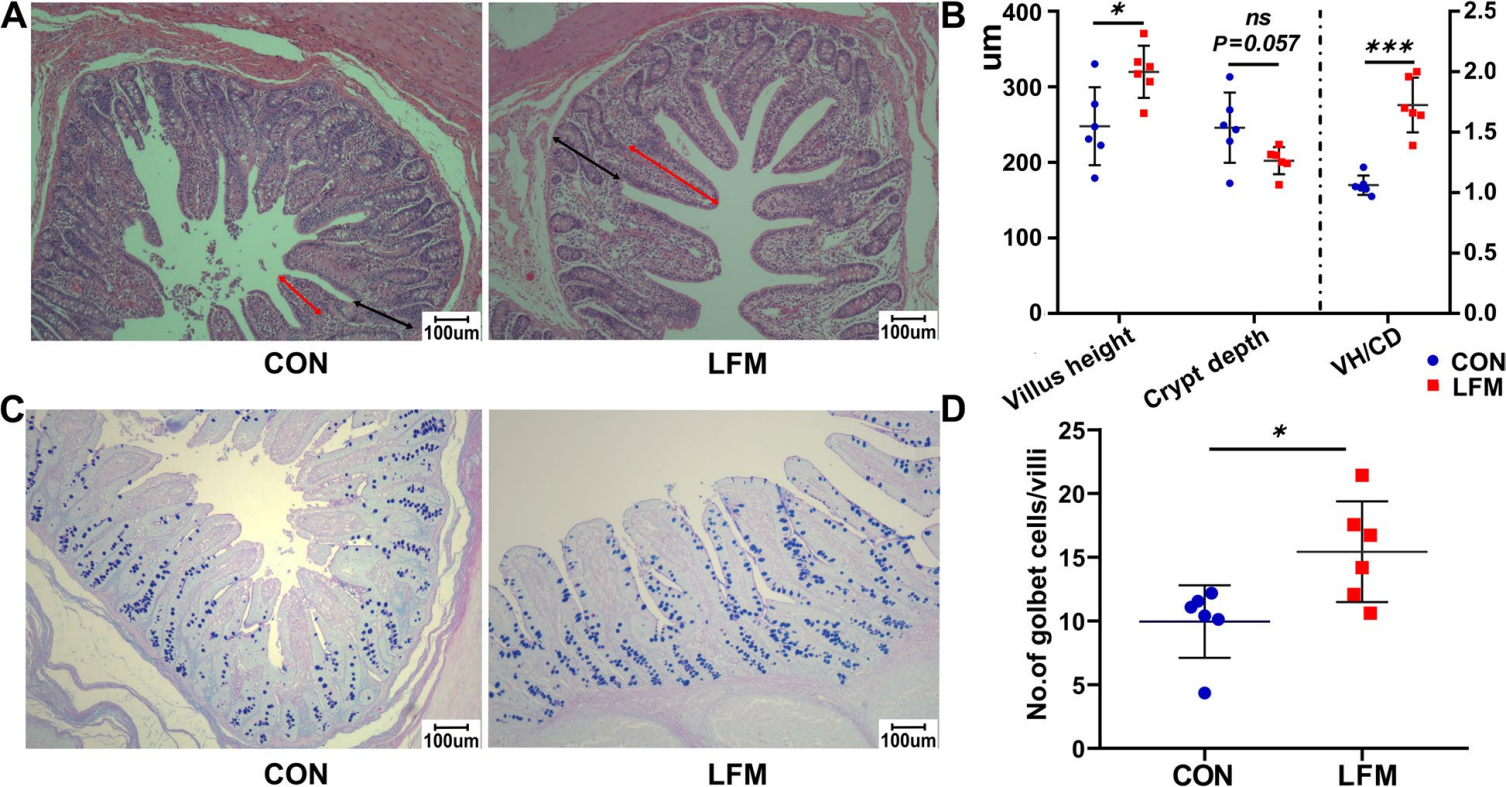
Effects of LFM on the morphology and goblet cell number of ileum. **A** Representative images of hematoxylin and eosin-stained ileal sections. Scale bar = 200 μm. **B** The VH, CD, and VH/CD in the ileum were quantified. **C** Representative images of alcian blue-stained ileal sections. Scale bar = 100 μm. **D** The number of AB-PAS positive goblet cells per ileal villi was quantified. Data are pooled from two groups of piglets (*n* = 6) and are mean ± SEM. *P < 0.05, **P < 0.01, ***P < 0.001

Investigation of intestinal morphology showed that LFM significantly increased villi height (*P* < 0.05) and the ratio of villi height to crypt depth (*P* < 0.01) compared with the CON group, while the crypt depth in the LFM group (*P* = 0.06) tended to be shorter than that of the CON group (Fig. 2A and B). Moreover, LFM supplementation significantly increased the number of goblet cells in per ileal villi (*P* < 0.05) of weaned piglets in comparison with that of the CON group (Fig. 2C and D). The levels of sIgA (*P* < 0.05), TNF-α (*P* < 0.05), and IL-10 (*P* < 0.01) in the LFM group were significantly increased compared with the CON group, while a significant decrease in the IL- 6 levels (*P* < 0.05) was observed in the LFM group (Table 2).

**Table 2:** Mucosal immune response in ileum to supplementary LFM^a^

Item	CON	LFM	*P*-value
sIgA, μg/mg pro	2.71 ± 0.25	3.41 ± 0.14	0.038
TNF-α, pg/mg pro	40.9 ± 2.30	47.48 ± 0.84	0.023
IL-10, pg/mg pro	15.4 ± 0.83	22.4 ± 1.04	0.000
IL-6, pg/mg pro	2.12 ± 0.07	1.79 ± 0.05	0.005

^a^Values are presented as mean ± SEM, *n* = 6; *CON*, a control group; *LFM*, a lactic acid bacteria-fermented formula milk supplementation group

### Microbiota composition in ileal content

**Fig. 3:**
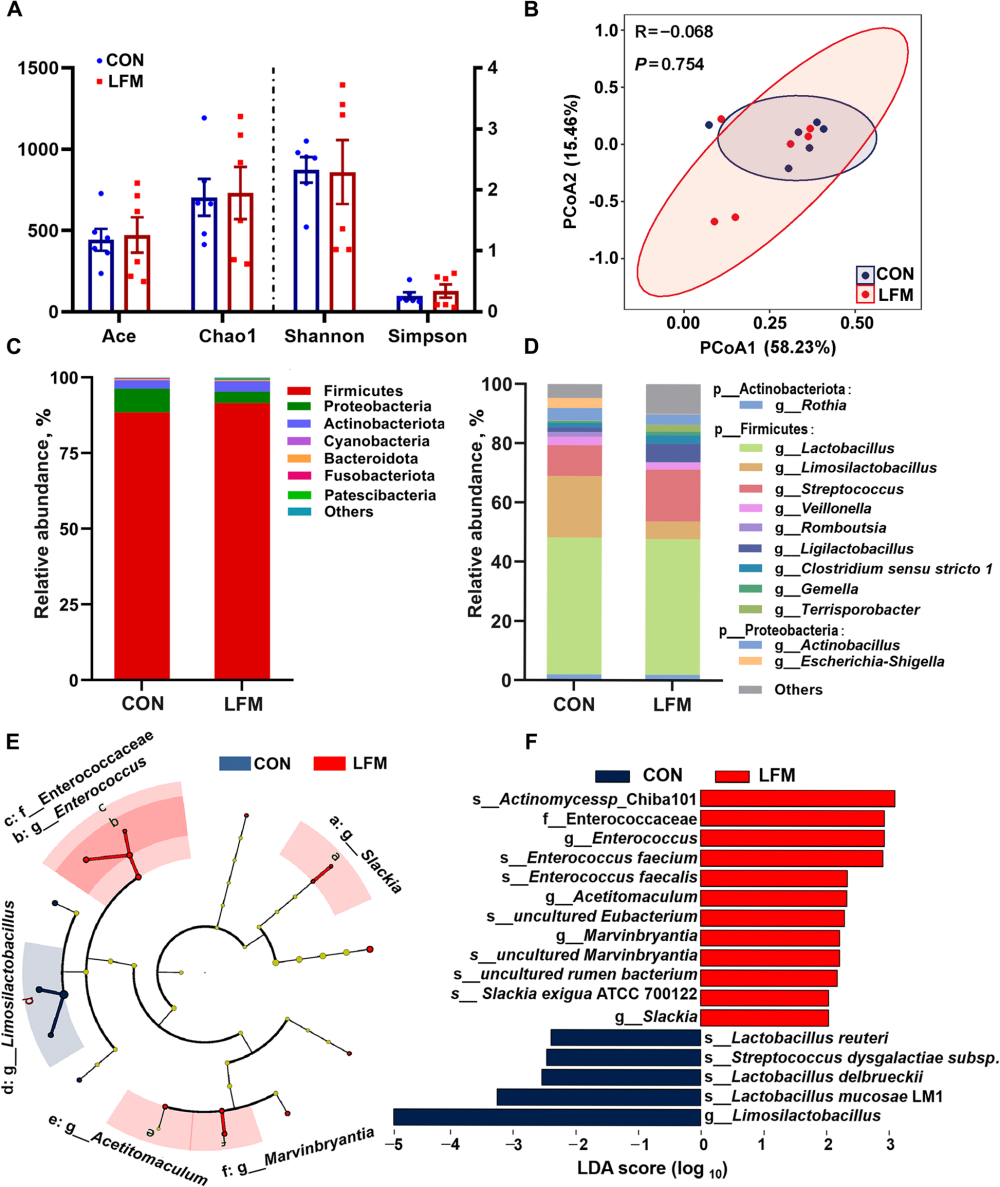
Effects of LFM supplementation on ileal microbiota. **A** The diversity of ileal microbiota in CON and LFM groups. **B** Principle coordinate analysis of ileum samples in the CON and LFM group. **C** Effects of lactic acid bacteria-fermented formula milk (LFM) supplementation on the phylum-level composition. **D** Effects of lactic acid bacteria-fermented formula milk (LFM) supplementation on the genus-level composition. **E** and **F** Taxonomic cladogram and LDA score plot generated from LEfSe of 16S rRNA gene amplification sequencing data (LDA score > 2, *P* < 0.1). Blue indicates enriched taxa in the CON group. Red indicates enriched taxa in the LFM group. Each circle's size is proportional to the taxon's abundance. Data are pooled from two independent pig groups (*n* = 6). Data are mean ± SEM

No differences in the richness estimators (Ace and Chao 1) and diversity indices (Shannon and Simpson) of ileal digesta microbiota were found between the two groups (Fig. 3A). According to the results from PcoA (Fig. 3B), the percentage of variation explained by PC1 and PC2 are 15.46% and 58.23%, respectively.

Furthermore, at the phylum level, the dominant bacterial groups were Firmicutes (CON group: 88.44%, LFM group: 91.50%), Proteobacteria (CON group: 7.87%, LFM group: 3.83%), and Actinobacteriota (CON group: 2.72%, LFM group: 3.46%), followed by Bacteroidota, Cyanobacteria, Fusobacteriota and Patescibacteria (Fig. 3C). At the genus level, the most dominant genus was *Lactobacillus* in both groups (CON group: 46.13%, LFM group: 45.72%). In addition, *Limosilactobacillus* (20.81%), *Streptococcus* (10.43%), *Actinobacillus* (4.21%), *Escherichia-Shigella* (3.41%), *Veillonella* (2.84%), *Rothia* (2.02%), *Romboutsia* (1.61%), *Ligilactobacillus* (1.57%), *Clostridium sensu stricto* 1 (1.50%) were the rest of abundant genera (> 1%) in the CON group (Fig. 3D). Different from the CON group, the remaining nine abundant genera (> 1%) in the LFM group were *Streptococcus* (17.54%), *Ligilactobacillus* (6.33%), *Limosilactobacillus* (6.01%), *Actinobacillus* (3.47%), *Clostridium sensu stricto* 1 (2.86% ), *Veillonella* (2.37%), *Terrisporobacter* (2.33%), *Rothia* (1.95%) and *Gemella* (1.22%).

Analysis with the LEfSe algorithm revealed that the CON group was characterized by *Limosilactobacillus* (LDA score = 4.90, *P* = 0.06) and related species such as *Lactobacillus mucosae LM1* (LDA score = 3.25, *P* = 0.06), *Lactobacillus delbrueckii* (LDA score = 2.54, *P* = 0.06) and *Lactobacillus reuteri* (LDA score = 2.39, *P* = 0.08). *Streptococcus dysgalactiae *subsp.* equisimilis*, a reported pathogen, was also enriched in the CON group (LDA score = 2.46, *P* < 0.05). However, in comparison with the CON group, several microbes belonging to the *Slackia* (LDA score = 2.04, *P* = 0.06), *Enterococcaceae* (LDA score = 2.93, *P* = 0.09), *Acetitomaculum* (LDA score = 2.33, *P* < 0.05) and *Marvinbryantia* (LDA score = 2.22, *P* = 0.08) were considered as the key species in the LFM group (Fig. 3E and F).

### PH, SCFA, and lactate concentrations in ileal content

As shown in Table 3, the pH value (*P* = 0.08) of the ileal digesta in the LFM group showed a downward tendency in comparison to the CON group, which was accompanied by an upward trend in concentrations of acetate (*P* = 0.07) and butyrate (*P* = 0.09) of the LFM-fed piglets. Nevertheless, there was no statistical difference in the levels of propionate and valerate between the two groups, but a significant increase in the lactate concentration (*P* < 0.05) was observed in the LFM group compared with the CON group.

**Table 3:** pH value and concentrations of SCFAs and lactate in ileal digesta^a^

Item	CON	LFM	*P*-value
pH value	7.07 ± 0.09	6.73 ± 0.15	0.082
Acetate, µmol/g	8.49 ± 0.38	9.40 ± 0.21	0.069
Propionate, µmol/g	1.91 ± 0.16	2.09 ± 0.25	0.568
Butyrate, µmol/g	0.78 ± 0.07	1.01 ± 0.10	0.091
Valerate, µmol/g	0.24 ± 0.02	0.23 ± 0.01	0.458
Lactate, µmol/g	30.43 ± 2.76	44.09 ± 4.04	0.019

^a^Values are presented as mean ± SEM, *n* = 6; *CON*, a control group; *LFM*, a lactic acid bacteria-fermented formula milk supplementation group

### Correlation analysis between microbiota and the measured parameters of ileum

**Fig. 4:**
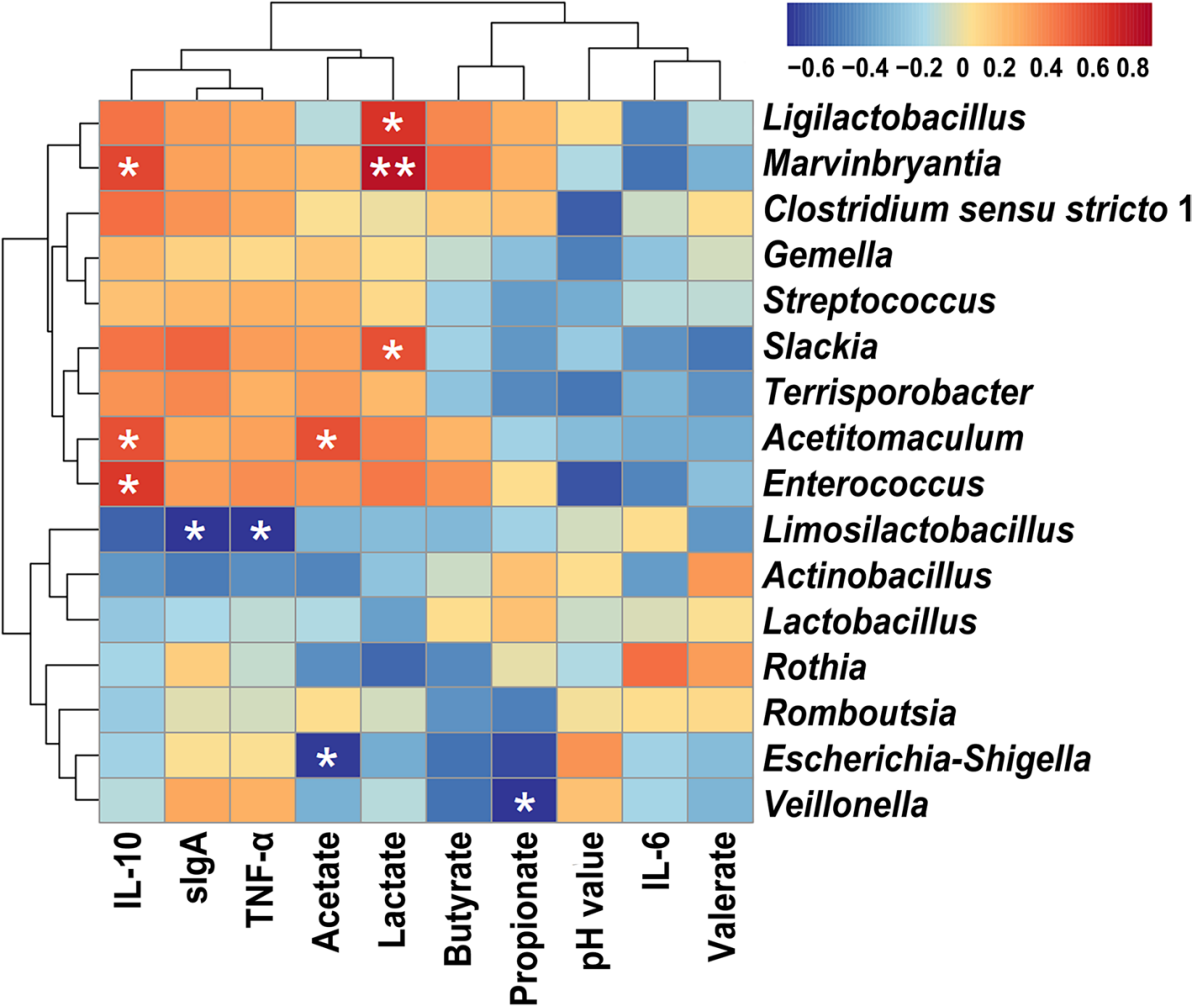
Heatmap of correlations between the ileal microbiota and the microbiota-associated metabolites, ileal pH value, and ileal sIgA and cytokines’ levels. The analysis is based on Pearson’s correlation coefficient. *P < 0.05; **P < 0.01

The Pearson’s correlation analysis (Fig. 4) showed significant negative correlations between the concentration of propionate and the relative abundances of *Veillonella* (*P* < 0.05). On the contrary, the relative abundances of *Ligilactobacillus* (*P* < 0.05), *Marvinbryantia* (*P* < 0.01), and *Slackia* (*P* < 0.05) had positive correlations with the level of lactate. The level of acetate was positively correlated with the relative abundance of *Acetitomaculum* (*P* < 0.05) but had a negative correlation with the relative abundance of *Escherichia-Shigella* (*P* < 0.05). The relative abundance of *Limosilactobacillus* showed a strong negative correlation with the concentrations of sIgA and TNF-α (*P* < 0.05). The abundances of *Marvinbryantia*, *Acetitomaculum*, and *Enterococcus* displayed a strong positive correlation with the level of IL-10 (*P* < 0.05). There was no obvious correlation between the pH value of ileum contents and changes in the relative abundances of ileal microbiota.

### Gene expression in ileal mucosa

**Fig. 5:**
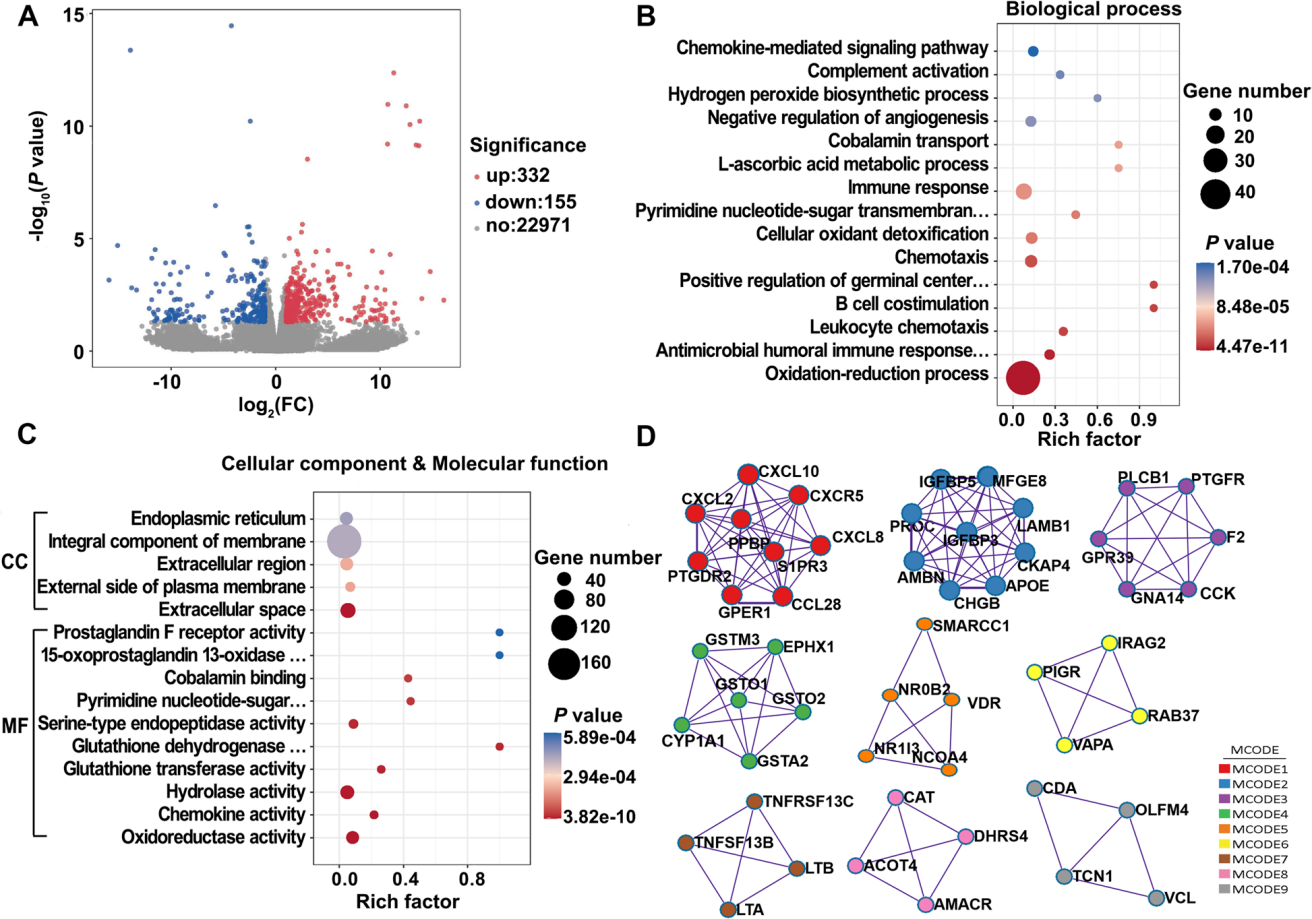
The GO enrichment analysis and protein–protein interactions network of DEGs. **A** Volcano plot of DEGs; fold-changes were calculated as CON treatment/LFM treatment. **B** The top 15 terms in the biological process category. **C** The top 5 terms in the cellular component category and the top 10 terms in the molecular function category. **D** Protein–protein interactions network among DEGs analysed by Metascape identifies nine protein communities. Results are based on RNA sequencing from 4 random samples in 6 biological replicates for each group

**Fig. 6:**
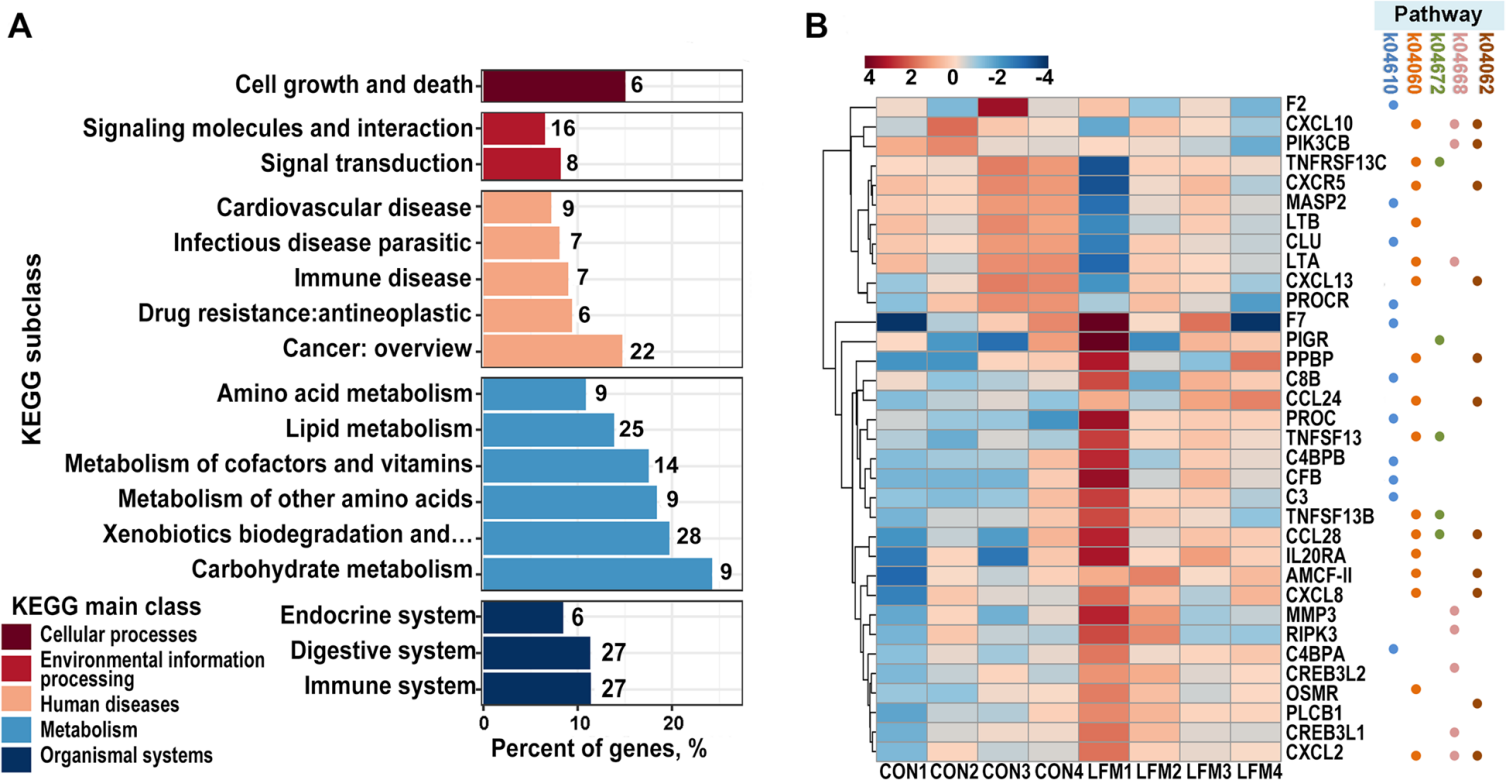
The KEGG enrichment analysis of DEGs. **A** Classification of significantly enriched KEGG pathway of DEGs. **B** Heatmap of 34 selected DEGs which are enriched in five immune-related pathways. Pathway, k04610: Complement and coagulation cascades; k04060: Cytokine-cytokine receptor interaction; k04672: Intestinal immune network for IgA production; k04668: TNF signalling pathway; k04062 Chemokine signalling pathway. DEGs, differentially expressed genes between the CON treatment and LFM treatment. Results are based on RNA sequencing from 4 random samples in 6 biological replicates for each group

The volcano plot of gene expression profiles showed that 487 DEGs (332 were up-regulated and 155 were down-regulated) were identified in the LFM group compared with the CON group (Fig. 5A). GO enrichment analysis was performed with those 487 DEGs between two groups. In the biological process category, GO terms associated with the oxidation–reduction process and antimicrobial humoral immune response mediated by antimicrobial peptide were predominant in the LFM group, followed by chemotaxis, cellular oxidant detoxification, and immune response (Fig. 5B). The top 5 GO terms observed in the cellular component process are mainly related to cell membranes, while the conspicuously enriched GO terms belong to molecular functions, including oxidoreductase activity, hydrolase activity, and serine-type endopeptidase activity (Fig. 5C). The interaction networks of proteins encoded by these DEGs identified nine protein communities (Fig. 5D). Notably, four of these protein communities were involved in immune-modulatory, including MCODE1, MCODE3, MCODE4, and MCODE7. In the KEGG pathway analysis, 34 pathways were significantly identified (Additional file 1: Table S3), divided into five main classes and fourteen subclasses according to the KEGG pathway database (Fig. 6A). Interestingly, the predominant KEGG subclass in organismal systems and metabolism was immune system and carbohydrate metabolism, respectively. As shown in Fig. 6B, there are five immune-related pathways enriched with a total of 34 differential genes, including C-X-C motif chemokine 10 (*CXCL10*), lymphotoxin alpha (*LTA*), and polymeric immunoglobulin receptor (*PIGR*). Finally, we verified several DGEs in the transcriptome by quantitative real-time PCR. The relative expression levels from real-time PCR and FPKM values from the transcriptomic data were presented as FC listed in Table 4. The result of real-time PCR was roughly consistent with that of the transcriptome, which confirmed the reliability of RNA-Seq data.

**Table 4:** The qPCR validation of the RNA-seq^a^

Genes^b^	RNA-seq^c^ (LFM vs. CON)		Real-time PCR^d^ (LFM vs. CON)	
	**FC**	***P*-value**	**FC**	***P*-value**
*TNFRSF13C*	0.46	0.037	0.36	0.048
*CXCL10*	0.50	0.024	0.42	0.026
*FABP1*	8.77	0.002	8.36	0.034
*LTA*	0.43	0.030	0.45	0.087
*LYZ1*	7.01	0.007	7.05	0.026
*PIGR*	6.02	0.013	6.14	0.042
*SLC5A8*	2.02	0.022	2.69	0.035
*TFF2*	5.06	0.001	5.02	0.004

^a^Data were presented as FC. The gene expressions were considered to be significantly altered when FC > 2 or < 0.5, *P* < 0.05

^b^*TNFRSF13C*: tumor necrosis factor receptor superfamily member 13C; *CXCL10*: C-X-C motif chemokine 10; *FABP1*: fatty acid binding protein 1; *LTA*: lymphotoxin alpha; *LYZ1*: lysozyme-like protein 1; *PIGR*: polymeric immunoglobulin receptor; *SLC5A8*: sodium-coupled monocarboxylate transporter; *TFF2*: trefoil factor 2

^c^Results based on RNA sequencing, *n* = 4

^d^Results based on real-time PCR, *n* = 6

## Discussion

LAB have been used to ferment foods for at least 4000 years. Previous evidence suggested that continuous consumption of the yogurt prepared with *Lactobacillus delbrueckii *subsp could stimulate the growth of specific indigenous *Lactobacillus* populations and modify the composition of intestinal *Lactobacillus* in pigs [25, 26]. Similarly, despite fasting for 12 h, we found that LFM supplementation resulted in an increased *Enterococcus* in the ileum, probably due to the successful colonization of *Enterococcus* from LFM. It is consistent with previous studies, where probiotic-enriched milk can be an effective carrier for the delivery of probiotics to the intestine [50]. It is well established that *Enterococcus* is one of the main representative genera of LAB. Most *Enterococcus* are considered gut commensals and commonly used in probiotic formulations [51, 52]. Strompfova et al. [53] demonstrated that feeding *Enterococcus faecium* to piglets aged 1–14 d can significantly reduce the number of *Escherichia-Shigella* in feces and the pH in the duodenum. However, in the present study, although we found that LFM could result in a downward tendency of pH value and the change of the list of dominant genera (> 1%) represented by the absence of *Escherichia-Shigella* in ileal digesta compared with the CON group, there were no significant differences in the relative abundance of *Escherichia-Shigella* between two groups. The inconsistent results with previous studies might be due to a limitation of our study that we analyzed too few individuals in each group. Even if the sample size of 6 provided adequate power (0.97) for the microbiological analysis based on a strong assumed effect size with ω^2^ = 0.12, we still could not overcome the individual differences and completely separate the clusters of ileal microbiota composition in the two groups by the PCoA analysis. However, three piglets from the LFM outside the CON group cluster showed an effect of LFM on microbiota diversity. In addition, in this study, only one piglet sample was randomly collected in each experimental replicate cage to analyze ileal microbiota and other intestinal parameters, which may low the statistical power and probably reduced the reliability of the results to a certain extent. Therefore, further investigation is still needed to evaluate the beneficial effects and potential mechanisms of LFM on intestinal health.

Our finding is consistent with earlier research that supplementation of yogurt prepared with *Enterococcus faecium* can increase the ADG of piglets [54], and our results further showed that the increased weaned weight gain might be due to the increased ADFI induced by the agreeable flavor of LFM. Contrary to expectations, no diarrhea occurred in either group, possibly due to the late weaning date of the piglets in this experiment. Therefore, whether LFM can alleviate diarrhea needs to be further explored through the feeding trial with earlier weaned or suckling piglets. We observed a significant improvement in ileal morphology, including the increased goblet cell count, higher villus height, and greater villus-crypt ratio in piglets of the LFM group compared with that of the CON group. However, the histological samples were fixed without cork plates in this experiment, wherefore a bias by the folding of tissue may affect the observation of intestinal morphology, so we measured thirty intact villi in each intestinal section to reduce the deviation. Transcriptomic profiling is widely used to identify critical genes and pathways. Thus, we randomly selected four piglets from each group for transcriptome analysis to find the critical differential expression genes, which were nearly as good as 6 replicates in terms of statistical power (> 0.90) but reduced the accuracy of logFC estimation to some extent. Compared with the CON group, the DEGs expression profile of LFM-fed piglets was involved in the immune system. Both oligosaccharides and LAB have been reported to affect the host immunity, which may be responsible for LFM-induced specific changes in gene expression.

As a goblet cell marker, the gene expression of *TFF2* of the LFM group was significantly increased than the CON group in both results of RNA-seq and RT-PCR. These results are supported by Lee et al. and Zhang et al., who demonstrated the role of lactic-acid-producing bacteria on mucosal barrier reconstruction, and the administration of LAB-type symbionts could significantly increase the expansion of ISCs, goblet cells, and Paneth cells [5, 55]. It is beneficial to intestinal health that ileal lactate level was significantly increased in LFM-fed piglets, most probably caused by the lactate-producing bacteria or the compound acidifier in LFM. Coincidently with the study by Viaud et al. [56], our results also demonstrated the synergistic immunogenic effect of *Enterococcus hirae*, which are positively correlated with the IL-10 levels in the ileal mucosa based on the correlation heatmap. Additionally, there was a higher tendency for the levels of acetate and butyrate in the LFM group than that in the CON group. A possible explanation is that more lactic acid was converted into SCFAs by lactic acid-utilizing bacteria, such as *Acetitomaculum* [57]. Of note, our sampling process took about 2 h, the impact on the acute stress response of euthanasia pretreatment and the difference in the last meal time may affect gut dynamics of SCFA production and absorption. In addition, we measured SCFA based on the wet weight of ileal digesta rather than dry matter, which may also cause inevitable experimental errors.

Transcriptome analysis suggested that LFM may promote the intestinal health of weaned piglets by regulating immune-related pathways, especially the Intestinal immune network for IgA production. Current studies have shown that sIgA is a major defense mechanism in protecting the intestinal epithelium from toxins and pathogens [58, 59]. Each sIgA molecule transported into the lumen must consume a polymeric immunoglobulin receptor (*PIGR*) molecule. Thus, the expression of *PIGR* is critical for the continuous supply of sIgA [60]. We observed that oral administration of LFM increased the gene expression of *PIGR* and intestinal sIgA level in weaning piglets. However, the specific mechanism of how LFM affects *PIGR* expression levels remains incompletely understood. Interestingly, a recent study demonstrated that porcine milk small extracellular vesicles (PM-sEVs) circ-XPO4 plays an important role in IPECJ2 cell or recipients through the absorption of miR-221-5p and activation of the *PIGR*, and eventually promote intestinal sIgA production in piglet [61]. In addition, previous research suggested that most of the beneficial activities from breast milk may be provided by its microbiota associated metabolites, and one clinical trial of *Lactobacillus paracasei* CBA L74-fermented formula indicated that Lactobacillus fermented milk has a nutritional value similar to breast milk in term of promoting intestinal sIgA level [62].

This study focused on the effects of the LFM on the ileal microbiota composition and mucosal immunity. Our findings indicate that supplementation of LFM could regulate the composition and metabolism of ileal microbiota and improve intestinal health and growth performance of weaned transition piglets. However, it remains to be further explored what bioactive ingredients (the probiotic LAB, microbiota-associated metabolites, or the combined effects) of the LFM play roles in alleviating weaning stress. In addition, the specific molecular mechanisms involved in regulating ileal mucosal immunity by LFM are worthy of further investigation.

## Conclusions

In conclusion, we found that continuous supplementation of LFM in the early weaning period of piglets could result in significant changes in microbial composition and transcriptomic profile in the ileum. More specifically, LFM could promote the colonization of probiotic *Enterococcus* and had the potential to inhibit the growth of *Escherichia-Shigella* in the ileum of piglets. LFM also altered microbiota metabolites, which may mediate the regulation of ileal immunity and the reconstruction of the intestinal barrier. This study reveals the possible mechanism underlying the effects of LAB-fermented formula milk on gut health in weaned piglets and provides substantial insight into the application of LFM in commercial pig farms.

## Supplementary Information

**Additional file 1: Table S1.** Dietary composition and nutrient levels. **Table S2.** Primer sequences are used for real-time PCR analysis. **Table S3.** KEGG pathway enrichment of DEGs.

## Data Availability

All data generated or analyzed during this study are available from the corresponding author on reasonable request.

## Abbreviations

AB-PAS: Alcian blue-periodic acid-shiff
ADFI: Average daily feed intake
ADG: Average daily gain
CD: Crypt depth
CFU: Colony forming units
Ct: Threshold cycle
DEGs: Differentially expressed genes
ELISA: Enzyme-linked immunosorbent assay
FC: Fold change
FDR: False discovery rate
F/G: Feed/gain ratio
FPKM: Fragments per kilobase per million mapped fragments
GO: Gene Ontology
HE: Hematoxylin and eosin
IL-6: Interleukin 6
IL-10: Interleukin 10
KEGG: Kyoto Encyclopedia of Genes and Genomes
LAB: Lactic acid bacteria
LDA: Linear discriminant analysis
LEfSe: Linear discriminant analysis effect size
OTUs: Operational taxonomic units
LFM: Lactic acid bacteria-fermented formula milk
PCoA: Principal coordinate analysis
PCR: Polymerase chain reaction
PIGR: Polymeric immunoglobulin receptor
SCFA: Short-chain fatty acid
SEM: Standard error of the mean
sIgA: Secretory immunoglobulin A
TFF2: Trefoil factor 2
TNF-α: Tumor necrosis factor-α
VH: Villus height
VH/CD: Villus-crypt ratio
