# Supplementary material for: Effects of lactic acid bacteria-fermented formula milk supplementation on ileal microbiota, transcriptomic profile, and mucosal immunity in weaned piglets
Source: J Anim Sci Biotechnol. 2022 Oct 6;13:113. doi: 10.1186/s40104-022-00762-8 (PMC9536082; doi:10.1186/s40104-022-00762-8)
Supplement: Supplementary file 1 — Additional file 1: Table S1. Dietary composition and nutrient levels. Table S2. Primer sequences are used for real-time PCR analysis. Table S3. KEGG pathway enrichment of DEGs. [file 40104_2022_762_MOESM1_ESM.docx]

Table S1. Dietary composition and nutrient levels

| **Ingredients, g/kg** | **Content** | **Nutrients levels, %^2^** | **Content** |
| --- | --- | --- | --- |
| Puffed corn flour | 240.0 | Dry matter | 86.7 |
| Broken rice noodles | 168.1 | Crude protein | 18.00 |
| Fish meal | 30.0 | Ether extract | 5.24 |
| Puffing of soybean | 50.0 | Crude fibre | 1.38 |
| Soybean oil | 6.5 | Ash | 5.32 |
| Fermented soybean meal | 80.0 | Ca | 0.76 |
| Stone powder | 8.0 | Digestible P | 0.31 |
| Calcium dihydrogen phosphate | 7.4 | Lysine | 1.5 |
| Flour | 50.0 | Methionine + Cystine | 0.82 |
| Sugar | 30.0 | Threonine | 0.90 |
| Glucose | 40.0 | Tryptophan | 0.25 |
| Milk powder | 100.0 | NaCl | 0.35 |
| Low protein whey powder | 100.0 | Valine | 0.93 |
| Soy protein concentrate | 40.0 | Isoleucine | 0.81 |
| High nucleotide yeast hydrolysate | 10.0 | Net energy, kcal/kg | 2435 |
| 4% Suckling pig premix^1^ | 40.0 | Digestible energy, kcal/kg | 3410 |
| Total | 1000 |  |  |

^1^The premix provided the following per kg of diets: VA 11,000 IU; VD_3_ 1000 IU; VE 16 IU; VK 11 mg; VB_1_ 0.6 mg; VB_2_ 0.6 mg; VB_6_ 1.5 mg; VB_12_ 0.03 mg; VB_4_ 800 mg; biotin 6 mg; nicotinic acid 10 mg; folic acid 0.8 mg; Fe (as ferrous sulfate) 165 mg; Zn (as zinc sulfate) 165 mg; Cu (as copper sulfate) 16.5 mg; Mn (as manganese sulfate) 30 mg; Co (as cobalt chloride) 0.15 mg; I (as potassium iodide) 0.25 mg; Se (as sodium selenite) 0.25 mg.

^2^Nutrient levels were calculated values

Table S2. Primer sequences used for real-time PCR analysis

| Genes^1^ | Sequence (5' to 3') | |
| --- | --- | --- |
| *TNFRSF13C* | F: GTGGTCTTGTCAGAGTCCTAC | R: GAAACGCAATTGAACAATCAGC |
| *CXCL10* | F: ACACAGGAGAAGCTAGAAATACC | R: TATTTTGAAGAGCACCCACTCA |
| *FABP1* | F: GGACATCGGAAATCGTGCAG | R: ACTGAACCACTGTCTTGACC |
| *LTA* | F: GAAGAAAGAAGAGGCGTTTCTG | R: ACGTGACAGTAGAGGTAGTAGA |
| *LYZ1* | F: GGTCTATGATCGGTGCGAGT | R: AACTGCTTTGGGTGTCTTGC |
| *PIGR* | F: AGCCAACCTCACCAACTTCC | R: CTGCTAATGCCCAGACCAC |
| *SLC5A8* | F: GGCACTCGTTTGTGAAGCTG | R: ATCCGCCCTCCCAAACATTC |
| *TFF2* | F: CCAAGAACCGGGTCAACT | R: CACTCCTCAGACTCTTGCG |

^1^*TNFRSF13C:* tumor necrosis factor receptor superfamily member 13C; *CXCL10:* C-X-C motif chemokine 10; *FABP1:* fatty acid binding protein 1; *LTA:* ymphotoxin alpha; *LYZ1：*lysozyme-like protein 1; *PIGR:* polymeric immunoglobulin receptor; *SLC5A8:* sodium-coupled monocarboxylate transporter; *TFF2:* trefoil factor 2

Table S3. KEGG pathway enrichment of DEGs^1^

| **KEGG subclass** | **Pathway name** | **Down-regulated genes** | **Up-regulated genes** | **-lgP** |
| --- | --- | --- | --- | --- |
| Amino acid metabolism | Cysteine and methionine metabolism | *\* | *AGXT2; AHCYL1; ENSSSCG00000040779; GCLC; GCLM* | 1.78 |
| Amino acid metabolism | Glycine, serine and threonine metabolism | *\* | *AGXT2; ENSSSCG00000040779; GLYCTK; ALDH7A1* | 1.43 |
| Cancer: overview | Chemical carcinogenesis | *\* | *ENSSSCG00000020706; ENSSSCG00000022724; ENSSSCG00000036274; GSTA2; GSTM3; GSTO1; GSTO2; EPHX1; CYP1A1; ENSSSCG00000009182; HSD11B1; CYP2C42* | 7.02 |
| Cancer: overview | Transcriptional misregulation in cancer | *MEF2C; PAX5; LYL1; LMO2; LDB1* | *MMP3; MET; PPARG; CXCL8; IGFBP3* | 1.37 |
| Carbohydrate metabolism | Ascorbate and aldarate metabolism | *MIOX* | *UGDH; ENSSSCG00000022724; ENSSSCG00000036274; ALDH7A1* | 3.74 |
| Carbohydrate metabolism | Pentose and glucuronate interconversions | *\* | *SORD; UGDH; ENSSSCG00000022724; ENSSSCG00000036274* | 2.33 |
| Cardiovascular disease | Fluid shear stress and atherosclerosis | *PIK3CB; MEF2C* | *NQO1; GSTA2; GSTM3; GSTO1; GSTO2; CTSV; PRKAA2* | 1.60 |
| Cell growth and death | Ferroptosis | *\* | *ACSL3; NCOA4; GCLC; GCLM; LPCAT3; SLC40A1* | 2.71 |
| Digestive system | Mineral absorption | *\* | *TRPV5; VDR; SLC40A1; SLC39A4; S100G; HEPH* | 2.95 |
| Digestive system | Pancreatic secretion | *ENSSSCG00000036013* | *CCK; ATP2B1; PLCB1; RAB27B; SLC12A2; CA2* | 1.53 |
| Digestive system | Salivary secretion | *\* | *TRPV5; ATP2B1; PLCB1; SLC12A2; DMBT1; LYZ* | 1.48 |
| Digestive system | Vitamin digestion and absorption | *\* | *LRAT; SLC19A3; CBLIF* | 1.45 |
| Digestive system | Carbohydrate digestion and absorption | *PIK3CB; ENSSSCG00000028277* | *ENSSSCG00000015691; ENSSSCG00000030013* | 1.36 |
| Drug resistance: antineoplastic | Platinum drug resistance | *PIK3CB* | *GSTA2; GSTM3; GSTO1; GSTO2; ABCC2* | 1.70 |
| Endocrine system | PPAR signaling pathway | *FADS2* | *MMP3; ACSL3; PPARG; FABP1; DBI* | 1.51 |
| Immune disease | Rheumatoid arthritis | *LTB* | *CTSV; MMP3; TNFSF13; TNFSF13B; AMCF-II; CXCL8* | 1.82 |
| Immune system | Complement and coagulation cascades | *F2; MASP2; PROCR; CLU* | *F7; ENSSSCG00000024914; PROC; C3; C8B; C4BPA; C4BPB* | 4.57 |
| Immune system | Intestinal immune network for IgA production | *TNFRSF13C* | *TNFSF13; TNFSF13B; CCL28; PIGR* | 1.99 |
| Immune system | Chemokine signaling pathway | *PIK3CB; CXCR5; ENSSSCG00000036445; CXCL10* | *PPBP; CXCL2; AMCFII; CCL28; PLCB1; CXCL8; ENSSSCG00000035736* | 1.55 |
| Infectious disease: parasitic | Amoebiasis | *PIK3CB* | *C8B; GNA14; LAMB1; VCL; PLCB1; CXCL8* | 1.58 |
| Lipid metabolism | Biosynthesis of unsaturated fatty acids | *FADS2* | *ACOT4; ELOVL6; HSD17B12* | 1.99 |
| Lipid metabolism | Steroid hormone biosynthesis | *HSD17B6* | *ENSSSCG00000022724; ENSSSCG00000036274; ENSSSCG00000011147; CYP1A1; CYP2D25; HSD17B12; HSD11B1* | 4.13 |
| Lipid metabolism | Fatty acid elongation | *\* | *ACOT4; ACAA2; ELOVL6; HSD17B12* | 2.11 |
| Lipid metabolism | Fatty acid degradation | *\* | *ACSL3; ACAA2; ENSSSCG00000009182; ALDH7A1* | 1.36 |
| Lipid metabolism | Glycolipid metabolism | *\* | *DGAT2; GLYCTK; GPAT3; AGPAT3; ALDH7A1* | 1.36 |
| Metabolism of cofactors and vitamins | Retinol metabolism | *HSD17B6* | *RDH5; LRAT; ENSSSCG00000022724; ENSSSCG00000036274; ALDH1A1; CYP1A1; DHRS4; RDH11; ENSSSCG00000009182; CYP2C42* | 6.25 |
| Metabolism of cofactors and vitamins | Folate biosynthesis | *\* | *ENSSSCG00000008309; ENSSSCG00000020706; ENSSSCG00000011147* | 1.40 |
| Metabolism of other amino acids | Glutathione metabolism | *CHAC1* | *PGD; GSTA2; GSTM3; GSTO1; GSTO2; GCLC; GCLM; ENSSSCG00000010056* | 4.53 |
| Signal transduction | TNF signaling pathway | *PIK3CB; LTA; CXCL10* | *MMP3; CXCL2; RIPK3; CREB3L1; CREB3L2* | 1.78 |
| Signaling molecules and interaction | Cytokine-cytokine receptor interaction | *LTB; CXCR5; TNFRSF13C; LTA; ENSSSCG00000036445; ENSSSCG00000036445; CXCL10* | *OSMR; IL20RA; PPBP; TNFSF13; TNFSF13B; CXCL2; AMCFII; CCL28; CXCL8; ENSSSCG00000035736* | 2.08 |
| Xenobiotics biodegradation and metabolism | Metabolism of xenobiotics by cytochrome P450 | *\* | *ENSSSCG00000020706; ENSSSCG00000022724; ENSSSCG00000036274; GSTA2; GSTM3; GSTO1; GSTO2; EPHX1; CYP1A1; ENSSSCG00000009182; HSD11B1* | 6.75 |
| Xenobiotics biodegradation and metabolism | Drug metabolism - cytochrome P450 | *\* | *FMO5; ENSSSCG00000022724; ENSSSCG00000036274; GSTA2; GSTM3; GSTO1; GSTO2; ENSSSCG00000009182* | 4.13 |
| Xenobiotics biodegradation and metabolism | Drug metabolism - other enzymes | *\* | *ENSSSCG00000022724; ENSSSCG00000036274; GSTA2; GSTM3; GSTO1; GSTO2; NME3; ENSSSCG00000002825* | 4.06 |

^1^*DEGs*, differentially expressed genes between the CON treatment and LFM treatment
